# The Potential Causal Association of Apolipoprotein A and B and Age-Related Macular Degeneration: A Mendelian Randomisation Study

**DOI:** 10.3390/biomedicines12122828

**Published:** 2024-12-12

**Authors:** Young Lee, Je Hyun Seo

**Affiliations:** 1 Veterans Medical Research Institute, Veterans Health Service Medical Center, Seoul 05368, Republic of Korea; lyou7688@gmail.com; 2 Department of Applied Statistics, Chung-Ang University, Seoul 06974, Republic of Korea

**Keywords:** age-related macular degeneration, apolipoprotein A, apolipoprotein B, smoking, Mendelian randomisation

## Abstract

Background/Objectives: Research has suggested a potential relationship between apolipoproteins A (ApoA) and B (ApoB) and age-related macular degeneration (AMD). This study explored the potential causal relationship between ApoA/ApoB levels and AMD/AMD subtypes using two-sample Mendelian randomisation (MR). Methods: We selected 308 single nucleotide polymorphisms (SNPs) for ApoA and 198 SNPs for ApoB from the UK Biobank data. Summary statistics for AMD were collected from the genome-wide association study of the FinnGen project. We performed two-sample MR to assess the causal effects of ApoA/ApoB on AMD and its subtypes. Potential confounders, including body mass index, C-reactive protein level, and smoking status, were assessed using a multivariable MR analysis. Results: ApoA showed a significant causal association with AMD (odds ratio [OR] = 1.14, 95% confidence interval [CI] = 1.05–1.25, *p* = 0.003) and was linked to both dry (*p* = 0.004) and wet (*p* = 0.025) AMD. ApoB showed a decreasing trend in dry AMD risk (*p* = 0.074), though not significant, and was not associated with overall or wet AMD. The multivariable MR analysis showed no significant association of ApoA with any AMD subtype (*p* > 0.05). ApoB decreased dry AMD risk (OR = 0.89, 95% CI = 0.80–0.99, *p* = 0.039), with trends for overall and wet AMD that were not significant (*p* = 0.070 and *p* = 0.091, respectively). Conclusions: These findings suggest that ApoB is associated with lower AMD risk, particularly for dry AMD. Further research is needed to clarify lipid biomarker’s role as AMD risk factors.

## 1. Introduction

Age-related macular degeneration (AMD) is the leading cause of blindness in the elderly, particularly in individuals aged > 60 years [1,2,3]. AMD is estimated to occur in 8.69% of the global population, affecting 196 million people in 2020, and this prevalence is expected to increase to 288 million by 2040 [2]. In addition, people living with AMD often require additional support, including caregiving services, vision aids, and rehabilitation, which further escalates their economic burden [4,5]. Despite the advances in treatment, AMD remains a major public health issue [6]. Although its pathogenesis is unclear, AMD is regarded as a complex, multifactorial disease with modifiable and nonmodifiable risk factors [6,7]. Advanced age, family history, inflammation, hypertension, hyperlipidaemia, body mass index (BMI), and cigarette smoking are well-known risk factors that have been consistently related to AMD in multiple studies [1,8,9,10,11,12,13]. AMD occurs when extracellular deposits, such as lipofuscin, minerals, and proteins, collectively known as drusen, accumulate in the outer retina, ultimately leading to photoreceptor degeneration and a loss of central vision. Two distinct manifestations occur in the late stages of AMD: the development of confluent areas of atrophy, involving photoreceptors and retinal pigment epithelium (RPE), known as geographic atrophy, and the growth of abnormal blood vessels in the macular region, referred to as neovascular (wet) AMD.

The relationship between lipids and AMD has been extensively studied, with mixed results [14,15,16,17]. Some studies have not found an association between serum lipid profiles and the risk of AMD [18,19]. In one study, elevated high-density lipoprotein-cholesterol (HDL-C) but not total cholesterol (TC) was associated with an increased risk of AMD [18]. No differences in TC, triglyceride (TG), phospholipid, HDL-C, and low-density lipoprotein-cholesterol (LDL-C) concentrations were observed between patients with AMD and controls [20]. However, other studies have suggested that higher serum HDL-C levels at baseline are associated with the geographic atrophy of AMD [21] and that HDL-C levels are linked to wet AMD [19]. Moreover, some studies have shown an inverse correlation between HDL-C levels and AMD risk, indicating that higher LDL-C levels are associated with an increased risk of advanced AMD [22]. These results are consistent with those of the Blue Mountain Eye Study, which demonstrated that increased HDL-C levels were inversely related to the incidence of late AMD (relative risk, 0.74; 95% confidence interval [CI], 0.56–0.99) [21]. Therefore, further research on serum lipid profiles and AMD is needed.

Apolipoproteins are important structural components of plasma lipoproteins that influence vascular biology and pathophysiology of atherosclerotic diseases by regulating lipoprotein metabolism. Apolipoprotein A (ApoA) is a crucial functional component of HDL particles that reverses cholesterol transport from peripheral tissues to the liver [23]. ApoA decreases blood cholesterol levels and prevents atherosclerosis [24]. As a component of good cholesterol, ApoA not only protects against heart diseases but also has profound effects on the central nervous system by preserving brain health [25]. In addition, ApoA has been reported as a prognostic marker in myeloproliferative disorders such as polycythaemia vera [25]. Apolipoprotein B (ApoB) is the main lipoprotein in LDL-C and a biomarker for cardiovascular diseases and has been evaluated in a previous study [26]. In addition, a previous study showed that ApoB is linked to type 2 diabetes, stroke, and longevity [27]. Studies on the connection between ApoA/ApoB and AMD are rare because ApoA or ApoB is not a primary test included in lipid panels in clinical practice. A previous study showed that ApoA and ApoB levels did not differ between patients with AMD and healthy controls [28]. One recent study demonstrated that the hazard ratio of ApoA for AMD was 1.40 (95% CI, 1.20–1.63) [29]. Therefore, the investigation of ApoA and ApoB, which have potential associations with serum HDL-C levels, is valuable for studying AMD.

In recent years, Mendelian randomisation (MR) has emerged as a useful research method for investigating putative causal relationships between risk factors and diseases using genetic variants in natural experiments [30,31]. MR is increasingly utilised because it can, to a certain degree, address a major limitation of observational studies: unmeasured confounding factors [32]. To minimise issues with certain confounding factors and support causal inference statements, MR uses genetic variation as an instrument. Several ophthalmological studies have examined the risk factors using MR [12,33,34,35]. A previous study used MR to show that higher HDL-C and ApoA levels increased the risk of AMD, whereas LDL-C and ApoB levels were associated with a decreased risk of atrophic AMD [36]. A study using summary statistics from the UK Biobank (UKB) as exposures and those from the International AMD Genomics Consortium (IAMDGC) and UKB as outcome data reported that elevated circulating HDL-C/ApoA levels increase the risk of early AMD [37]. More scalable research can be conducted by adding the analysis of wet AMD, a type of late AMD, in addition to early AMD in combined multivariable MR to control for confounders, such as C-reactive protein, smoking, and BMI, which are risk factors for AMD.

Recently, the FinnGen project has begun to provide a variety of data, allowing for more in-depth research. Considering the importance of lipids in the mechanism of AMD and the inconsistent findings regarding the risk factors for AMD of HDL-C and LDL-C in previous studies [14,15,16,17], it would be meaningful to analyse these key molecules, ApoA and ApoB, using MR. Further attention is required because a recent phenome-wide MR study on AMD demonstrated various metabolites and lipoproteins [38]. Hence, in this study, we explored the potential causal effects of ApoB and ApoB on AMD (subtypes: dry AMD and wet AMD) using a two-sample MR and multivariable MR with C-reactive protein, BMI, and smoking as cofounders, using summary statistics from the UKB and FinnGen.

## 2. Materials and Methods

### 2.1. Study Design

The Institutional Review Board of the Veterans Health Service Medical Centre approved the study protocol (IRB No. 2023-12-030; 11 January 2024). The requirement for informed consent was waived due to the retrospective nature of this study. This study was conducted in compliance with the principles of the Declaration of Helsinki.

**Figure 1:**
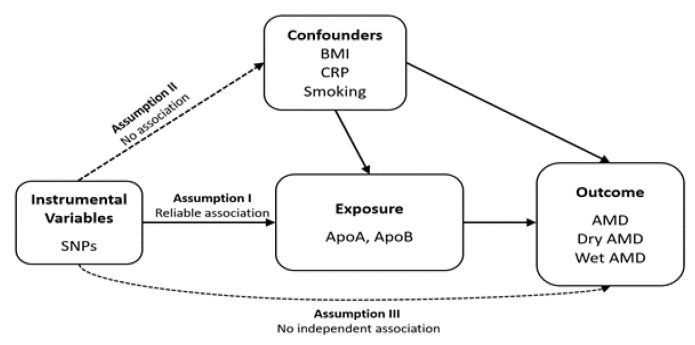
A schematic diagram of the Mendelian randomisation study design. BMI, body mass index; CRP, C-reactive protein; SNP, single-nucleotide polymorphism; ApoA, apolipoprotein A; ApoB, apolipoprotein B; and AMD, age-related macular degeneration. Solid lines represent the presence of an association, while dashed lines indicate the absence of an association.

A schematic representation of the study design is shown in Figure 1. Data were obtained from summary statistics from the UKB (*n* = 364,987 for ApoA and *n* = 399,003 for ApoB). To assess the causal effects of ApoA and ApoB on AMD, multivariable MR analysis incorporated additional UKB datasets for potential confounders: 419,163 participants for BMI; 400,094 participants for C-reactive protein; and 418,817 participants for smoking (43,192 cases vs. 375,625 controls). For the outcome analysis, we used the summary dataset from the FinnGen project for AMD (*n* = 357,849; 8913 cases vs. 348,936 controls), dry AMD (*n* = 257,107; 6065 cases vs. 251,042 controls), and wet AMD (*n* = 257,125; 4848 cases vs. 252,277 controls). Details of the sources of the summary data are provided in Table 1.

### 2.2. Selection of the Genetic Instrumental Variables

The study used single-nucleotide polymorphisms (SNPs) associated with exposure at the GWAS significance threshold (*p* < 5.0 × $10^{-8}$) as instrumental variables (IVs). To maintain the independence of each IV, SNPs were selected after pruning for linkage disequilibrium (LD; r^2^ < 0.001, clumping distance = 10,000 kb). Data from the 1000 Genomes Phase III European study were used as a reference to calculate the LD during the clumping process. The F-statistic, used to assess potential weak instrument issues, indicates that values above 10 suggest no evidence of weak instrument bias [41].

### 2.3. Mendelian Randomisation

We employed a two-sample MR design to estimate the causal effects of ApoA and ApoB on AMD and its subtypes. Our primary analysis was performed using the inverse variance-weighted (IVW) method with multiplicative random effects, which is considered the most efficient method when all IV assumptions are met [41,42,43]. To ensure robustness, we applied the weighted median method [44], MR-Egger regression (with and without adjustment via the simulation extrapolation [SIMEX] method) [45,46], and MR pleiotropy residual sum and outlier (MR-PRESSO) method [47]. The weighted median approach provides reliable estimates even if up to 50% of the instruments are invalid [44]. MR-Egger regression allows for the estimation of causal effects in the presence of pleiotropy by accounting for a nonzero intercept, which represents the average horizontal pleiotropic effects [45]. When the ‘no measurement error’ assumption is violated (*I*^2^ < 90%), MR-Egger regression with SIMEX is used to correct for potential bias [46]. MR-PRESSO detects and corrects horizontal pleiotropy by identifying outliers and recalculating the IVW estimates after their removal [47]. Heterogeneity was assessed using Cochran’s Q statistic for the IVW method and Rücker’s Q′ statistic for the MR-Egger method [42,48]. Significant heterogeneity suggests potential pleiotropy [42,49]. We further evaluated directional horizontal pleiotropy using the MR-PRESSO global test [47]. A *p*-value < 0.05 in Cochran’s Q, Rücker’s Q′, or the MR-PRESSO global test indicated potential pleiotropy.

Since previous studies have reported multivariable MR results using serum lipid markers [36,37,38], major risk factors for AMD other than lipid markers were selected in this study. Based on previous research results, confounding variables were selected based on known AMD risk factors [50]. Several studies have reported that a higher BMI is a risk factor for AMD, an age-related eye disease [51,52]. Previous reports have shown that increased systemic C-reactive protein (CRP) levels are risk factors for AMD [12,53,54]. One study reported that smoking is associated with higher risk of wet AMD (odds ratio [OR] 1.26, 95% CI = 1.13–1.40, *p* < 0.001) [55]. Substantial evidence has demonstrated that smoking is a major risk factor for AMD [56,57,58]. Given these results, we selected BMI, CRP level, and smoking as potential confounders in the multivariable MR analysis to assess the effects of ApoA and ApoB on AMD. In the multivariable MR analysis, we employed both the multivariable IVW [30] and MR-Egger [59] approaches to adjust for these confounders. The results are reported as ORs with 95% CIs. All MR analyses were conducted using the TwoSampleMR, Mendelian randomisation, and Simex packages in R version 3.6.3 (R Core Team, Vienna, Austria). Power calculations were conducted using mRnd (https://cnsgenomics.com/shiny/mRnd/, accessed on 20 November 2024) [60], assuming that the proportions of variance in ApoA and ApoB explained by IVs were 11% and 7.7%, respectively. These values were calculated by determining the proportion of variance explained by each individual IV and then summing them.

## 3. Results

### 3.1. Genetic Instrumental Variables

In our MR study, we identified 308 SNPs for ApoA and 198 SNPs for ApoB as IVs, all of which were genome-wide significant and mutually independent. The F-statistic values for all selected SNPs exceeded 10, with mean values of 130.62 for ApoA and 155.68 for ApoB, indicating strong instrument strength. Detailed information on the IVs used in this study is provided in Supplementary Table S1. Our data provided sufficient statistical power (>80%) for a causal analysis of ApoA and ApoB in AMD and its subtypes (Supplementary Table S2). The assumption of no measurement error was supported by *I*^2^ values of 97.54% for ApoA and 98.34% for ApoB (Table 2). Heterogeneity among the IVs was assessed using Cochran’s Q test from the IVW method and Rücker’s Q’ test from the MR-Egger analysis, both of which indicated significant heterogeneity (*p* < 0.05; Table 2), suggesting potential pleiotropy. The MR-PRESSO global test results for horizontal pleiotropy were also significant (*p* < 0.05; Table 2). However, the MR-Egger intercepts, which were close to zero, did not indicate horizontal pleiotropy (*p* > 0.05; Table 2). Given the low power of the MR-Egger method in detecting pleiotropy, the MR-PRESSO method is recommended for addressing potential pleiotropy [61].

### 3.2. Mendelian Randomisation for the Effects of ApoA and ApoB on AMD/AMD Subtypes

We investigated the potential causal relationships between ApoA, ApoB, and AMD, including its subtypes, using 308 SNPs for ApoA and 198 SNPs for ApoB as IVs in the univariable MR analysis. The outliers were identified and corrected using the MR-PRESSO method to enhance the reliability of the causal estimates. For ApoA, six outliers were identified for AMD, three for dry AMD, and six for wet AMD. Similarly, for ApoB, seven outliers were identified for AMD, three for dry AMD, and six for wet AMD. Detailed information on these outliers is provided in Supplementary Table S1. After removing outliers, the IVW estimates were recalculated to obtain the corrected MR-PRESSO estimates.

**Figure 2:**
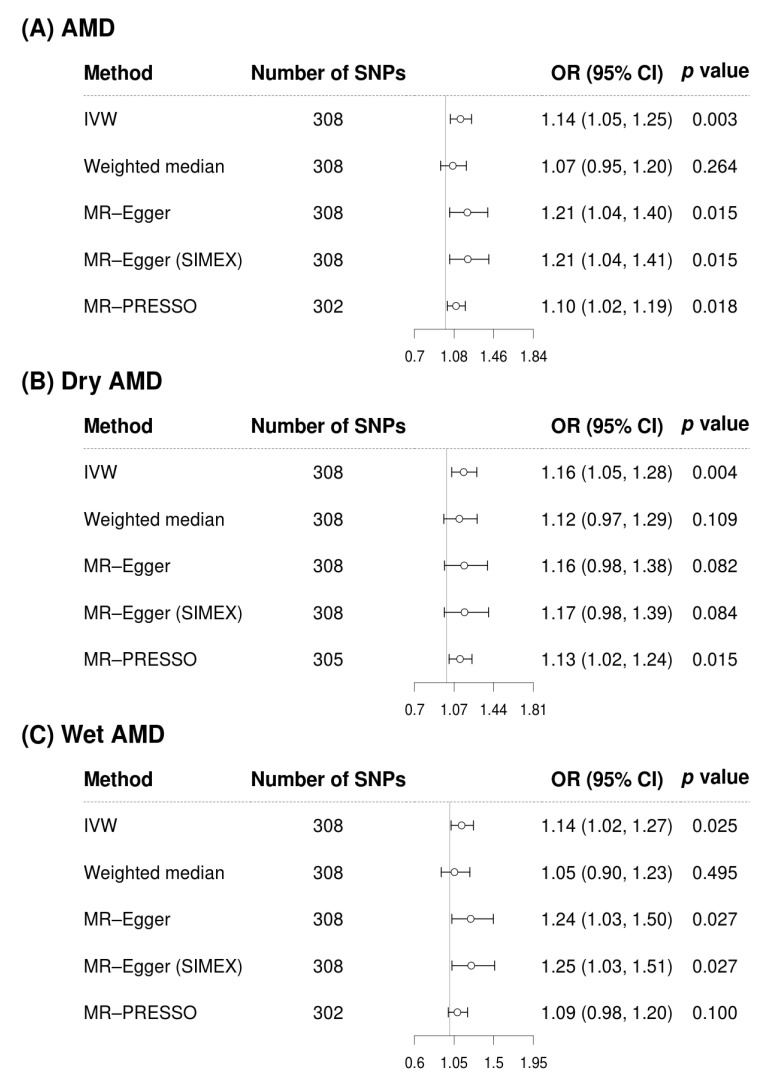
Forest plots of the causal associations of apolipoprotein A with AMD and AMD subtypes. AMD, age-related macular degeneration; SNP, single-nucleotide polymorphism; OR, odds ratio; CI, confidence interval; IVW, inverse-variance weighted; MR, Mendelian randomisation; SIMEX, simulation extrapolation; PRESSO, pleiotropy residual sum and outlier.

ApoA increased the risk of AMD across several methods (Figure 2A), including IVW (OR = 1.14, 95% CI = 1.05–1.25, *p* = 0.003), MR-Egger (OR = 1.21, 95% CI = 1.04–1.40, *p* = 0.015), MR-Egger (SIMEX) (OR = 1.21, 95% CI = 1.04–1.41, *p* = 0.015), and MR-PRESSO (OR = 1.10, 95% CI = 1.02–1.19, *p* = 0.018). Similar results were observed for the AMD subtypes. Specifically, ApoA was associated with an increased risk of dry AMD in both the IVW (OR = 1.16, 95% CI = 1.05–1.28, *p* = 0.004) and MR-PRESSO (OR = 1.13, 95% CI = 1.02–1.24, *p* = 0.015) analyses (Figure 2B). For wet AMD, ApoA also showed an increased risk according to the IVW (OR = 1.14, 95% CI = 1.02–1.27, *p* = 0.025), MR-Egger (OR = 1.24, 95% CI = 1.03–1.50, *p* = 0.027), and MR-Egger (SIMEX) (OR = 1.25, 95% CI = 1.03–1.51, *p* = 0.027) methods (Figure 2C), although the MR-PRESSO method did not show statistical significance (OR = 1.09, 95% CI = 0.98–1.20, *p* = 0.100).

**Figure 3:**
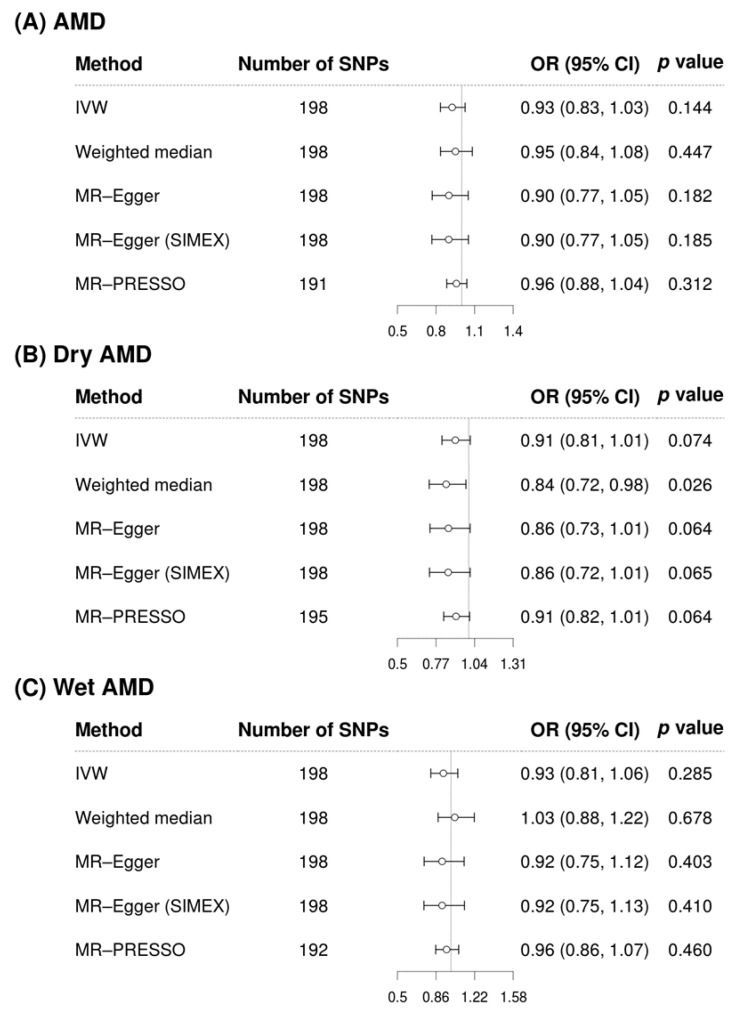
Forest plots of the causal associations of apolipoprotein B with AMD and AMD subtypes. AMD, age-related macular degeneration; SNP, single-nucleotide polymorphism; OR, odds ratio; CI, confidence interval; IVW, inverse-variance weighted; MR, Mendelian randomisation; SIMEX, simulation extrapolation; PRESSO, pleiotropy residual sum and outlier.

Although ApoB did not show any significant association with AMD (all *p* > 0.05, Figure 3A), a potential protective effect of ApoB on dry AMD was suggested by the IVW (OR = 0.91, 95% CI = 0.81–1.01, *p* = 0.074), weighted median (OR = 0.84, 95% CI = 0.72–0.98, *p* = 0.026), MR-Egger (OR = 0.86, 95% CI = 0.73–1.01, *p* = 0.064), MR-Egger (SIMEX) (OR = 0.86, 95% CI = 0.72–1.01, *p* = 0.065), and MR-PRESSO (OR = 0.91, 95% CI = 0.82–1.01, *p* = 0.064) methods (Figure 3B). No significant association was found between ApoB and wet AMD (*p* > 0.05; Figure 3C).

**Figure 4:**
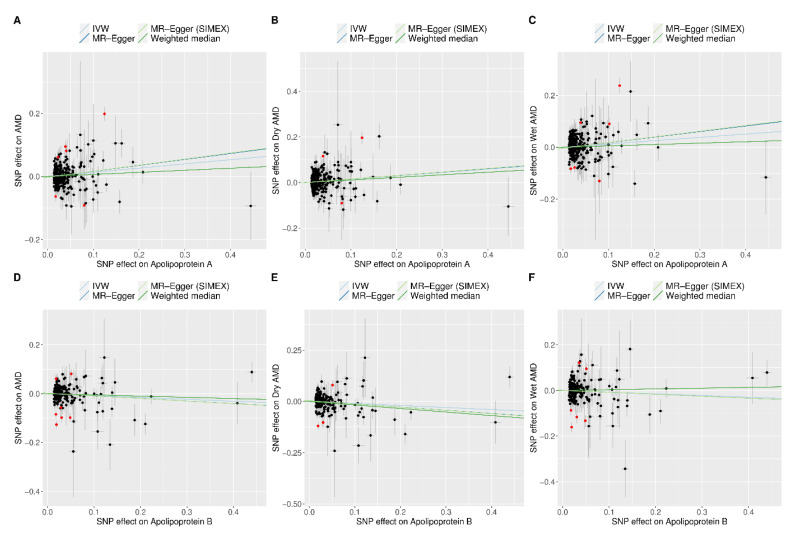
Scatter plots of MR tests assessing the effects of apolipoproteins A and B on AMD and AMD subtypes. Scatter plot indicating the effects of apolipoproteins A on AMD (**A**), dry AMD (**B**), and wet AMD (**C**) as well as those of apolipoproteins B on AMD (**D**), dry AMD (**E**), and wet AMD (**F**). Light blue, dark blue, light green, and dark green regression lines represent the IVW, MR-Egger, MR-Egger (SIMEX), and weighted median estimates, respectively. The slope of each line reflects the causal effect estimated by the corresponding method. Each dot corresponds to a SNP, with the x-axis representing the association between the SNP and the exposure, and the y-axis representing the association between the SNP and the outcome. Red dots indicate outliers identified by the MR-pleiotropy residual sum and outlier analysis. AMD, age-related macular degeneration; IVW, inverse-variance weighted; MR, Mendelian randomisation; SIMEX, simulation extrapolation; SNP, single-nucleotide polymorphism.

Figure 4 presents scatter plots showing the effects of SNPs on ApoA and ApoB and their respective effects on AMD, dry AMD, and wet AMD. A positive correlation between ApoA levels and AMD was observed, which was consistent for both dry and wet AMD. For ApoB, no notable slopes were observed for overall AMD or wet AMD; however, a negative association with dry AMD was evident.

In a multivariable MR analysis with BMI, CRP, and smoking as potential confounders, ApoA did not show any significant effect on the risk of AMD, dry AMD, or wet AMD (*p* > 0.05; Table 3). In contrast, ApoB was associated with a marginally reduced risk of AMD (OR = 0.92, 95% CI = 0.83–1.01, *p* = 0.070 in IVW; OR = 0.92, 95% CI = 0.83–1.01, *p* = 0.068 in MR-Egger). Notably, similar to the univariable MR results, ApoB significantly reduced the risk of dry AMD (OR = 0.89, 95% CI = 0.80–0.99, *p* = 0.039 in both IVW and MR-Egger methods). ApoB also showed a protective trend against wet AMD (OR = 0.90, 95% CI = 0.80–1.02, *p* = 0.091 in IVW; OR = 0.90, 95% CI = 0.80–1.02, *p* = 0.094 in MR-Egger), although these results were not statistically significant. Neither BMI nor CRP levels were significantly associated with AMD or its subtypes (*p* > 0.05). However, smoking increased the risk of AMD (OR = 1.24, 95% CI = 1.05–1.46, *p* = 0.009 in IVW; OR = 1.24, 95% CI = 1.05–1.46, *p* = 0.010 in MR-Egger) and dry AMD (OR = 1.28, 95% CI = 1.06–1.55, *p* = 0.012 in IVW; OR = 1.28, 95% CI = 1.06–1.55, *p* = 0.012 in MR-Egger). No significant association was found between smoking and wet AMD (*p* > 0.05).

## 4. Discussion

Our study demonstrated a possible causal association of ApoA and increased risk of AMD, dry AMD, and wet AMD using univariable MR and that ApoB levels decreased the risk of dry AMD in both univariable and multivariable MR. Numerous studies have examined the association between lipids and AMD, with varying results. A significant correlation has been reported between high total serum cholesterol levels and wet AMD [62]. In addition, a meta-analysis found that HDL-C levels were positively correlated with AMD rates but not with late AMD and that high total serum cholesterol levels protected against dry AMD. In addition, LDL-C levels showed no effect on late AMD but seemed to be protective against dry AMD [14], which is consistent with the findings of this study. A recent study reported that ApoA was positively associated with the risk of dry AMD (OR = 2.04, *p* < 0.05), whereas ApoB had a protective effect against dry AMD (OR = 0.56, *p* < 0.05) [37], in line with our findings. ApoB is a critical structural protein of atherogenic lipoproteins, and excess LDL-L can cause fatty deposits (plaques) in arterial walls and hardening and scarring of blood vessels, resulting in atherosclerosis and consequent coronary heart diseases, carotid diseases, and peripheral vascular diseases [63]. Thus, several GWAS of ApoA and ApoB have been performed [64,65,66]. These results help improve the understanding of mechanistic pathways and diseases. Different views exist on the mechanism by which AMD is linked to lipid metabolism. Further research on the mechanisms underlying AMD is required to elucidate this link.

Although ApoB appeared to protect against dry AMD in univariable and multivariable MR analyses, its borderline significance needs to be explored more thoroughly. One possible hypothesis is that the AMD-wide and dry/wet AMD are heterogeneous [67], considering that wet AMD is accompanied by neovascular changes. Therefore, wet AMD may be caused by factors other than lipid metabolism. One phenotype of dry AMD is the presence of drusen and the accumulation of histochemically detectable neutral lipids in the RPE–Bruch’s membrane complex. In dry AMD, drusen involves the accumulation of oxidised lipoproteins, various forms of cholesterol, lipid-rich and structurally unstable lesions, and inflammation-driven downstream events, which are strikingly parallel to ApoB-instigated disease in the arterial intima. Research on intraocular sources of lipoprotein particles, biological processes that drive lipoprotein production, and lipoprotein particles as a source of extracellular cholesterol has been increasing [68,69,70]. Although the mechanisms underlying our findings are not fully understood, our results are supported by our current hypotheses. The RPE offloads undesirable lipids from phagocytosis of the outer segments at the apical surface to the systemic circulation, whereas lipoproteins in the plasma transport lipophilic essentials to the basolateral surface of the RPE. However, a number of factors, such as age-related decline in RPE endo-/exo-cytotic function and atherosclerotic impairment of lipid exchange across the Bruch’s membrane–choriocapillaris endothelium, cause this lipid exchange to become unbalanced in AMD [71]. Except for the ApoA content, these lipoproteins resemble LDL-C particles and contain both ApoA and ApoB in addition to a high amount of esterified cholesterol, which may act as a barrier to lipid transport through the aging retina. This results in the vulnerability of the Bruch’s membrane to lipid deposits and drusen. The formation of AMD lesions is thought to share mechanisms with atherosclerotic plaque formation. In atherosclerosis, apolipoprotein B100 lipoproteins trapped within the arterial wall initiate a cascade of pathological events, including innate immune system-mediated inflammation [72]. These processes may be accompanied by oxidative stress and inflammation, which contribute to the progression of wet AMD to wet AMD [72]. Hence, ApoA levels were associated with the risk of AMD/wet AMD, indicating higher HDL-C levels. The results of earlier studies support these findings, which showed that higher levels of ApoA, the major apolipoprotein in HDL-C particles, increase the risk of early AMD, with OR 2.04 (95% CI: 1.50–2.77) [37]. As HDL-C and LDL-C, which are related to ApoA and ApoB, respectively, constitute the total body cholesterol, they might explain the protective effect of ApoB. However, it is difficult to explain the protective effect of ApoB against dry AMD alone; thus, further research is required. Recent studies have shown that ApoE subtypes, which are biomarkers for Alzheimer’s disease, are associated with AMD [73,74]. Similar to ApoA and ApoB, ApoE is a major apolipoprotein in peripheral lipid metabolism in the central nervous system, and it is present in drusen and basal laminar deposits in macular areas [75]. There is convergent research on the relationship between ApoE and AMD [76], and this is also an area that requires further research.

The main strength of our study lies in the use of a large cohort dataset, which allowed us to explore the potential causal relationship between ApoA/ApoB and AMD/AMD subtypes within a single ethnic group using an MR analysis. In addition, our study suggests that in addition to lipoproteins, apolipoproteins are closely related to the risk of dry AMD and can potentially act as biomarkers. However, this study has several limitations. First, we did not have access to individual-level data, which prevented us from accounting for numerous confounding factors while using summary statistics in the two-sample MR approach. This also limits our ability to investigate the potential nonlinear relationship between ApoA/ApoB and AMD. Second, the methods used to validate the MR assumptions do not offer complete validation. Violations of these assumptions could lead to biased conclusions, necessitating a careful interpretation of the findings. In particular, significant heterogeneity was observed in Cochran’s Q and Rücker’s Q tests, suggesting potential pleiotropy. While the MR-Egger intercept results did not indicate strong evidence of directional pleiotropy, it is important to note that the MR-Egger intercept had limited power to detect pleiotropy, especially in cases of subtle effects. To address this, we used the MR-PRESSO method to correct for horizontal pleiotropy by identifying and removing outlier IVs. Nevertheless, residual pleiotropy or unaccounted heterogeneity cannot be entirely excluded, and these limitations should be considered when interpreting our results. Third, all participants in this study were of European descent, and further research is required to determine whether the results can be generalised to other ethnic groups. According to a previous study using a genomic analysis, AMD risk was higher in non-European groups, suggesting the need for further research [77]. Finally, the MR framework assumes that genetically predicted biomarker changes have lifelong linear effects on AMD risk. Consequently, the short-term effects and potential nonlinear relationships between these biomarkers remain unclear. Furthermore, based on the findings of a previous study [50], we included potential confounders closely linked to lipid metabolism, using a multivariable MR analysis.

## 5. Conclusions

Our study demonstrated a causal association between ApoA and an increased risk of AMD, whereas ApoB was associated with a decreased risk of dry AMD. Further research is required to address the significance of lipid biomarkers as risk factors for AMD.

## Figures and Tables

**Table 1:** Summary statistics of data sources.

Traits	Data Source	No. of Participants	Population	No. of Variants	Reference
ApoA	UKB	364,987	European	22,798,735	
ApoB	UKB	399,003	European	23,001,507	[39]
BMI	UKB	419,163	European	23,111,080	
CRP	UKB	400,094	European	23,008,369	
Smoking	UKB	418,817 (43,192 cases + 375,625 controls)	European	22,122,417	
AMD	FinnGen	357,849 (8913 cases + 348,936 controls)	European	20,169,869	
Dry AMD	FinnGen	257,107 (6065 cases + 251,042 controls)	European	20,165,949	[40]
Wet AMD	FinnGen	257,125 (4848 cases + 252,277 controls)	European	20,165,938	

ApoA, apolipoprotein A; ApoB, apolipoprotein B; BMI, body mass index; CRP, C-reactive protein; UKB, UK Biobank; AMD, age-related macular degeneration.

**Table 2:** Heterogeneity and horizontal pleiotropy of instrumental variables.

Exposure	Outcome				Heterogeneity		Horizontal Pleiotropy				
								MR-Egger Intercept		MR-Egger (SIMEX) Intercept	
		N	F	*I*^2^ (%)	*p* *	*p* #	*p* †	β (SE)	*p*	β (SE)	*p*
ApoA	AMD	308	130.62	97.54	<0.001	<0.001	<0.001	−0.002 (0.003)	0.372	−0.003 (0.003)	0.358
ApoB		198	155.68	98.34	<0.001	<0.001	<0.001	0.002 (0.003)	0.627	0.002 (0.003)	0.624
ApoA	Dry AMD	308	130.62	97.54	<0.001	<0.001	<0.001	−0.000 (0.003)	0.952	−0.000 (0.003)	0.935
ApoB		198	155.68	98.34	<0.001	<0.001	<0.001	0.003 (0.003)	0.373	0.003 (0.003)	0.368
ApoA	Wet AMD	308	130.62	97.54	<0.001	<0.001	<0.001	−0.004 (0.003)	0.267	−0.004 (0.003)	0.256
ApoB		198	155.68	98.34	<0.001	<0.001	<0.001	0.001 (0.004)	0.869	0.001 (0.004)	0.870

N, number of instruments; F, mean F statistic; MR, Mendelian randomisation; SIMEX, simulation extrapolation; β, beta coefficient; SE, standard error; ApoA, apolipoprotein A; ApoB, apolipoprotein B; AMD, age-related macular degeneration. * Cochran’s Q test from the inverse-variance weighted. # Rücker’s Q’ test from MR-Egger. † MR-pleiotropy sum of residuals and outlier global test.

**Table 3:** The multivariable MR results of the effects of ApoA, ApoB, BMI, CRP, and smoking on AMD and its subtypes.

	AMD				Dry AMD				Wet AMD			
	IVW		MR-Egger		IVW		MR-Egger		IVW		MR-Egger	
	OR 95% CI	*p*	OR 95% CI	*p*	OR 95% CI	*p*	OR 95% CI	*p*	OR 95% CI	*p*	OR 95% CI	*p*
ApoA	1.01 0.92–1.11	0.852	1.01 0.92–1.11	0.843	1.01 0.90–1.13	0.837	1.01 0.90–1.13	0.839	0.98 0.86–1.11	0.736	0.98 0.86–1.11	0.732
ApoB	0.92 0.83–1.01	0.070	0.92 0.83–1.01	0.068	0.89 0.80–0.99	0.039	0.89 0.80–0.99	0.039	0.90 0.80–1.02	0.091	0.90 0.80–1.02	0.094
BMI	0.99 0.86–1.14	0.898	0.98 0.84–1.15	0.812	0.98 0.83–1.15	0.794	0.98 0.82–1.18	0.836	0.99 0.83–1.18	0.898	0.99 0.81–1.22	0.960
CRP	1.05 0.94–1.17	0.396	1.04 0.91–1.19	0.577	1.07 0.94–1.22	0.326	1.07 0.91–1.25	0.399	1.08 0.93–1.24	0.321	1.08 0.91–1.29	0.369
Smoking	1.24 1.05–1.46	0.009	1.24 1.05–1.46	0.010	1.28 1.06–1.55	0.012	1.28 1.06–1.55	0.012	1.16 0.94–1.44	0.164	1.16 0.94–1.44	0.162

MR, Mendelian randomisation; ApoA, apolipoprotein A; ApoB, apolipoprotein B; BMI, body mass index; CRP, C-reactive protein; AMD, age-related macular degeneration; IVW, inverse-variance weighted; OR, odds ratio; CI, confidence interval.

## Data Availability

The datasets used and/or analysed in the current study are available from the Pan-UK Biobank (https://pan.ukbb.broadinstitute.org/downloads/index.html, accessed on 17 June 2024) and FinnGen project (https://finngen.gitbook.io/documentation/data-download, accessed on 4 November 2023).

## Supplementary Materials

The following supporting information can be downloaded at https://www.mdpi.com/article/10.3390/biomedicines12122828/s1, Table S1: List of single-nucleotide polymorphisms used as instrumental variables in univariable MR analysis. Table S2: Power calculations for MR analysis.
